# Acute Kidney Injury following Cardiopulmonary Bypass: A Challenging Picture

**DOI:** 10.1155/2021/8873581

**Published:** 2021-03-09

**Authors:** Dianxiao Liu, Baohui Liu, Zhenxing Liang, Zhi Yang, Fangjian Ma, Yang Yang, Wei Hu

**Affiliations:** ^1^Department of Cardiac Surgery, Binzhou Medical University Hospital, 661 Huanghe 2nd Road, Binzhou 256603, China; ^2^Key Laboratory of Resource Biology and Biotechnology in Western China, Ministry of Education, Faculty of Life Sciences, Northwest University, 229 Taibai North Road, Xi'an 710069, China; ^3^Department of Cardiothoracic Surgery, The First Affiliated Hospital of Zhengzhou University, 1 Jianshe East, Zhengzhou 450052, China; ^4^PLA Unit 93307, 118 Honghu North Street, Shenyang 110141, China; ^5^Xi'an Key Laboratory of Cardiovascular and Cerebrovascular Diseases, Xi'an No.3 Hospital, School of Life Sciences and Medicine, Northwest University, 10 Fengcheng Three Road, Xi'an 710021, China

## Abstract

Recent studies have recognized several risk factors for cardiopulmonary bypass- (CPB-) associated acute kidney injury (AKI). However, the lack of early biomarkers for AKI prevents practitioners from intervening in a timely manner. We reviewed the literature with the aim of improving our understanding of the risk factors for CPB-associated AKI, which may increase our ability to prevent or improve this condition. Some novel early biomarkers for AKI have been introduced. In particular, a combinational use of these biomarkers would be helpful to improve clinical outcomes. Furthermore, we discuss several interventions that are aimed at managing CPB-associated AKI, may increase the effect of renal replacement therapy (RRT), and may contribute to preventing CPB-associated AKI. Collectively, the conclusions of this paper are limited by the availability of clinical trial evidence and conflicting definitions of AKI. A guideline is urgently needed for CPB-associated AKI.

## 1. Introduction

Cardiopulmonary bypass (CPB) is a form of extracorporeal circulation that temporarily replaces the function of the heart and lungs during surgery to maintain the circulation of blood and oxygen in the patient, which has benefited thousands of patients since its introduction nearly 60 years ago [1, 2]. The 2017 Heart Disease and Stroke Statistics from the American Heart Association reported outcomes for approximately 400,000 patients undergoing cardiac surgical procedures each year, and more than 80% of these procedures were performed using CPB [3]. However, CPB is not a benign procedure, and a number of associated problems remain, including hemolysis [4], capillary leak syndrome [5], and acute kidney injury (AKI) [6–8]. AKI occurs in 18.2% to 30% of patients who undergo CPB surgery [7, 9–11] and is an important predictor of morbidity and mortality after cardiac surgery [12–15].

AKI is defined as the rapid deterioration of kidney function within 48 hours after the initiating event [16, 17] and exerts a separate independent effect on the risk of death [18, 19]. Generally, AKI could be caused by a decrease in the renal blood flow, renal inflammation, or pigment nephropathy resulting from any reason. Septic shock and CPB surgery are the two of the most common factors that contribute to AKI [20]. In patients undergoing CPB, AKI is associated with poor outcomes, prolonged hospital stays, increased mortality, and stroke [6–8, 21, 22]. In particular, CPB-associated AKI was associated with an 8.2-fold increase in in-hospital mortality [11].

Until a decade ago, a uniform definition of AKI was lacking, creating problems in comparing published results. In 2007, the AKI Network (AKIN) modified the risk, injury, failure, loss, end-stage renal disease (RIFLE) criteria established in 2004 by including an absolute change in the serum creatinine (sCr) level, which also decreased the time over which AKI was to be diagnosed from 7 days to 48 hours [16, 17]. The recent 2012 Kidney Disease: Improving Global Outcomes (KDIGO) criteria combined RIFLE and AKIN, providing clear guidelines for the timing of AKI ascertainment and severity staging based on changes in sCr levels and urine output [23]. Based on accumulating evidence from meta-analyses, a consistency of prognostic estimates exists across AKI definitions. Notably, if the expected hospital stay is less than 7 days, the AKIN definition may be the most suitable [7].

Renal replacement therapy (RRT) is necessary for treating CPB associated-AKI [24], which is experienced by approximately 1% to 2.9% of all patients who undergo CPB surgery [9, 21]. Additionally, the use of RRT is a marker of early mortality and long-term mortality [25], similar to CPB-associated RRT [7, 21, 26]. The thirty-day mortality rate of patients with CPB-associated AKI who require RRT is 42% [21]. An estimate of the variation in the risk factors associated with clinical outcomes is needed to contribute to the prevention of CPB-associated AKI [7]. The detection of biomarkers is useful to prevent CPB-associated AKI [27]. We must improve our understanding of the mechanisms involved in CPB-associated AKI to prevent this disease and to develop comprehensive interventions for managing CPB-associated AKI when it occurs.

The focus of this review is to provide a comprehensive evaluation of CPB-associated AKI. First, the risk factors for CPB-associated AKI are summarized. Then, we present a review of the methods currently used to manage AKI following CPB, including RRT and other interventions. Finally, potential directions for future CPB-associated AKI are discussed. Collectively, the compiled information should serve as a comprehensive repository of the evidence that is currently available in this area, which should aid in the design of future studies. This review should contribute to the prevention and management of CPB-associated AKI.

## 2. Risk Factors for Acute Renal Dysfunction following Cardiopulmonary Bypass

Generally, the relative risk of AKI has decreased by 8% per year beginning in 2000 [28], and AKI-associated mortality is also decreasing [29]. Several risk factors have been identified to predict the development of CPB-associated AKI. The risk factors are classified as patient-related and procedure-related factors, and a comprehensive understanding of these factors and AKI should further contribute to our ability to control CPB-associated AKI.

### 2.1. Patient Conditions

An older age is an independent risk factor for developing AKI [30, 31]; for example, an age > 70 years is an independent risk factor for postoperative AKI, with a relative risk ranging from 2 to 2.232 [95% confidence interval (CI), 1.326-3.757; *P* < 0.005] [32, 33]. Female sex is another established independent patient-related risk factor for the development of CPB-associated AKI. Most meta-analyses have revealed that women are more likely than men to develop AKI postoperatively [odds ratio (OR), 1.21; 95% CI, 1.09-1.33; *P* < 0.001] [34]. A smoking history is also an independent factor (OR, 2.008; 95% CI, 1.144-3.524; *P* = 0.0151) [34]. A left ventricular ejection fraction (LVEF) < 35% was shown to be an independent risk factor for postoperative AKI (OR, 1.25; 95% CI, 1.01-2.2; *P* = 0.01) [33]. Furthermore, controlled clinical trials revealed a high risk of the development of AKI in children with congenital heart disease [26, 35, 36], with reported incidences ranging from 29% to 86% [36].

The presence of any extent of preoperative renal dysfunction (which is defined as a sCr level ≥ 1.2 mg/dL) increases the risk of CPB-associated AKI compared with the very low risk (<2%) for a patient with normal renal function under the same conditions [37, 38]. The preoperative estimated glomerular filtration rate (eGFR) is the best indicator of postoperative renal dysfunction. Based on several lines of evidence, a baseline eGFR < 60 mL/min/1.73 m^2^ is associated with an increased risk of CPB-associated AKI [38–42]. Furthermore, diabetes is also suggested to be independently associated with AKI [43], which may result from the deterioration of already borderline renal function [44, 45]. A decreased eGFR and borderline renal function may therefore be collectively associated with the incidence of CPB-associated AKI. Patient-related factors must be carefully evaluated to obtain a better risk stratification. Preventive approaches, such as glucose control, are suggested to be administered to reduce the burden of AKI and prevent CPB-associated AKI [33, 43]. Thoughtful and individualized decisions regarding the treatment of patients with multiple risk factors are necessary.

### 2.2. Creatinine

An elevated preoperative sCr level is the most significant predictive risk factor for postoperative AKI following CPB described to date [46, 47], as also evidenced by higher peak postoperative creatinine levels within 48 h of arrival in the intensive care unit (ICU) associated with persistent AKI [32]. The risk of AKI is increased 4.8-folds for each 1 mg/dL increase in the sCr level [38]. The patient's risk for postoperative dialysis after AKI reaches 10–20% with a baseline creatinine concentration of 2.0-4.0 mg/dL and approximately 25% when the baseline creatinine concentration is greater than 4.0 mg/dL [48]. In addition to inducing the development of AKI, preoperative creatinine levels greater than 2.5 mg/dL increase the risk of mortality and prolong the length of hospital stay following CPB surgery [49]. Collectively, these data indicate the value of monitoring and focusing on increased sCr levels throughout the perioperative period.

### 2.3. Genetic Polymorphisms

The clinical predictors and biochemical markers that have been identified as being associated with the development of AKI only partially explain the individual risk [20]. Another tool for predicting the risk of AKI and improving individualized patient care is focused on identifying the genetic risk factors that might be involved in the development of AKI. Several genetic polymorphisms have been identified to play roles in the occurrence and progression of AKI after cardiac surgery with CPB. As shown in the study by Leaf et al. [50], patients with a longer allele genotype in the heme oxygenase-1 (HO-1) gene (*HMOX1*) promoter exhibited an increased risk of postoperative AKI after cardiac surgery with CPB (OR, 1.26; 95% CI, 1.05-1.503; *P* = 0.01). This finding is consistent with heme toxicity as a pathogenic feature of cardiac surgery-associated AKI, suggesting the potential of HO-1 as a therapeutic target in the future. Additionally, Popov et al. [51] examined SNP rs1617640 in the promoter of the *erythropoietin* (*EPO*) gene using DNA sequencing and found that the risk allele rs1617640 (T) plays a role in the development of AKI after cardiac surgery with CPB. Patients with the TT risk allele produced increased concentrations of EPO and required more frequent acute RRT. A patient's ability to produce more EPO may be associated with thromboembolic events and therefore affects morbidity and mortality after CPB. Furthermore, Stafford-Smith et al. [52] identified two novel susceptibility loci (chr3p21.6 and BBS9) for AKI after cardiac surgery with CPB. These data provide candidate regions for future genetic research on cardiac surgery-associated AKI and may ultimately contribute to improvements in preoperative screening and the development of novel prevention and intervention options to decrease AKI and associated morbidity and mortality. Despite the substantial progress, formidable challenges regarding the widespread clinical application of genetic testing remain due to its high cost and time-consuming process.

### 2.4. Hemoglobin Concentration

Based on accumulating evidence, the hemoglobin concentration measured during CPB surgery is associated with the incidence of AKI after CPB surgery. According to a study by Haase et al. [11], a decreased hemoglobin concentration during CPB surgery is an independent risk factor for AKI, with an effect cut-off value of <9 g/dL (<5.6 mmol/L) (OR, 1.16 per 1 g/dL decrease; 95% CI, 1.05-1.31; *P* = 0.018), which is not altered by systemic arterial oxygen saturation and pressure values. Other studies also support that the low hemoglobin concentration even within the normal range, as well as the nadir hemoglobin level, is associated with increased incidence of CPB-associated AKI [53, 54]. Strategies that improve the hemoglobin concentration, such as the conservative use of red blood cell (RBC) transfusion, are recommended, since circulating free iron-mediated nephrotoxicity with hemolysis and free hemoglobin are likely to lead to AKI in patients undergoing cardiac surgery with CPB. However, the volume of transfused RBCs represents a specific additional risk factor if this treatment is administered to patients with hemoglobin levels > 8 g/dL (>5 mmol/L). Therefore, future studies should further investigate if modified blood conservation strategies or the restriction of RBC transfusion to patients with a hemoglobin concentration < 8 g/dL (<5 mmol/L) will improve renal outcomes. Is increasing the hemoglobin concentration with an EPO supplement and other methods more beneficial for patients with decreased hemoglobin concentrations?

### 2.5. Hemodilution

Hemodilution is another independent risk factor for developing renal injury (including AKI), with the lowest predictive cut-off value being a hematocrit < 24% [37]. A multivariate analysis revealed a 7% increase in the relative risk of AKI per 1% decrease in the nadir hematocrit value during CPB [28]. Hemodilution-induced renal injury was exacerbated when CPB was prolonged by the use of intraoperative packed RBC transfusions [37], suggesting a conflict between maintaining adequate hydration and using extended diuretic therapy and avoiding hemodilution [35]. Improvements in oxygen delivery conditions may contribute to solving this problem [55]. Moreover, the hemodilution-related AKI risk has been limited by many improvements to CPB technology that have been proposed in the past decade, including the redesign of the circuit to minimize the volume, the use of retrograde autologous priming, and the management of intraoperative fluid administration [28].

Notably, Svenmarker et al. [56] observed a decrease in the sCr concentration following CPB-induced hemodilution, which significantly hampered the creatinine-based diagnosis of AKI. Thus, the use of sCr levels to monitor renal function during CPB should be employed with caution to avoid underestimating the risk of AKI.

### 2.6. Oxygen Delivery

According to an increasing number of studies, the kidneys may suffer from an imbalance between the amount of oxygen that is available and the amount of oxygen they need during CPB [57]. As shown in the study by Ranucci et al. [58], the best predictor for AKI and peak postoperative sCr levels was the lowest oxygen delivery during CPB, with a critical threshold of <272 mL/min/m^2^. The effects of decreased hemoglobin concentrations and hemodilution on the incidence of AKI during CPB are potentially attributed to a decrease in the oxygen carrying capacity [57]. A decreased oxygen carrying capacity related to hemodilution might be compensated by increasing CPB oxygen delivery using an adequately increased pump flow. Interestingly, in patients with severe anemia (<25th percentile for the lowest hemoglobin level), the independent effect of hypotension (>75th percentile of the area under the curve for MAP < 50 mmHg) on AKI was pronounced [OR, 3.36 (95% CI 1.34-8.41); *P* = 0.010] [11]. After correcting for the need for transfusions, only the lowest level of oxygen delivery remained an independent risk factor for AKI during CPB [58]. Excursions of mean arterial blood pressure that were below the limit of autoregulation were independently associated with AKI, but not the absolute mean arterial blood pressure [43]. Inadequate oxygen delivery during CPB was associated with lactate production, and hyperlactatemia appeared when oxygen delivery decreased below 260 mL/min/m^2^ under normothermic conditions [59]. Thus, monitoring the cerebral oximetry index using near-infrared spectroscopy signals may represent a novel method for precisely guiding procedures aimed at maintaining a mean arterial blood pressure target during CPB, which further reflects the level of renal perfusion [43, 60]. In addition, pump flow should be coupled with hematocrit monitoring to avoid a decrease below the critical level of oxygen delivery. However, the extent to which the dose for oxygen delivery can be decreased remains to be estimated.

Different interventions aimed at preserving oxygen delivery during CPB have been reported to exert beneficial effects on goal-directed perfusion applications, and the incidence of AKI has been reduced from 5.8% to 3.1% [28]. Four elements of the formula for oxygen delivery under CPB, including pump flow, hemoglobin, oxygen saturation, and arterial oxygen tension, can be carefully monitored and properly modified, thus promoting the likelihood of adequate oxygen delivery [61]. The ability to maintain oxygen delivery and satisfy all these components requires cooperation and coordination among the members of the cardiac surgical team. In addition, Bennett et al. [62] compared the average oxygen delivery during bypass for a miniaturized CPB and for a conventional CPB circuit and did not observe differences in the average oxygen delivery. The association between oxygen delivery and postoperative changes in plasma creatinine levels was clear in both groups. However, further studies are needed to determine whether a particular cohort of patients benefits (or are put at risk) from each method of CPB. Furthermore, oxygen preconditioning has been shown to prevent nephropathy in patients [63] and AKI [64], renal ischemia/reperfusion injury [65], and nephrotoxicity in rats [66]. Accordingly, the use of hyperbaric oxygen therapy and oxygen preconditioning may be beneficial for preventing AKI following CPB.

### 2.7. Use of Angiographic Examinations

Contrast agent has been suggested to induce AKI in patients undergoing coronary angiography or percutaneous coronary interventions [44]. Ranucci et al. [28] showed that a policy restricting angiographic examination on the day of operation reduced the AKI rate from 4.8% to 3.7%. Performing cardiac surgery on the day of cardiac catheterization and using a higher dose of contrast agent were independently associated with an increased risk of postoperative AKI [67]. Interestingly, the administration of contrast to cyanotic patients with CHD within the 48 h prior to CPB was not an additional risk factor for developing AKI [46]. In conclusion, the angiographic examination may be not associated with an increased AKI rate. However, delaying cardiac surgery for more than 24 h after exposure to contrast agents (when feasible) and minimizing the use of contrast agents may significantly decrease the incidence of postoperative AKI in patients undergoing CPB surgery.

### 2.8. Time on CPB

Time on CPB was associated with the risk of developing AKI requiring dialysis [41, 68–71] In one study, after adjusting for confounders, the association between the time on CPB and AKI requiring dialysis lost its statistical significance [71]. Thus, an accurate risk assessment might be more important than the time on CPB in predicting the occurrence of AKI requiring dialysis. Nonetheless, the CPB time should be minimized to the greatest extent possible to reduce the risk of AKI following CPB in cyanotic patients with congenital heart disease [35].

**Figure 1:**
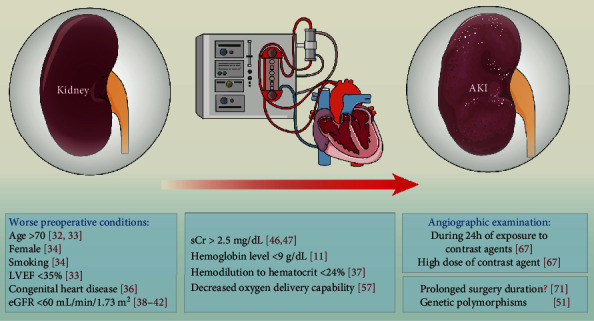
Risk factors for CPB-associated AKI. In addition to a set of independent risk factors that has been widely accepted and included in some predictive models, other risk factors are related to CPB-associated AKI, including genetic polymorphisms, a decreased hemoglobin concentration, hemodilution, and a decreased oxygen delivery capability. AKI: acute kidney injury; LVEF: left ventricular ejection fraction; eGFR: estimated glomerular filtration rate; sCr: serum creatinine.

Collectively, these risk factors could be converted into a simple, accurate, and reliable bedside risk tool, which should promote improved clinician-patient discussions about risks of CPB-associated AKI. High-risk patients (defined as an age > 70 years, female gender, smoking history, left ventricular ejection fraction < 35%, worse borderline renal function, increased sCr levels around the perioperative period, decreased hemoglobin concentration, hemodilution, and genetic susceptibility) should be targeted for renal protective strategies, and clinicians should focus on preventing the occurrence of AKI following CPB in these patients (Figure 1 and Table 1). Taking these factors into account, improvements to CPB technologies have been proposed to limit the risk of occurrence of AKI. Long et al. have proposed that perfusion techniques may be associated with the incidence of AKI [8]. Furthermore, a set of interventions that are mainly aimed at limiting and improving these risk factors may be effective at reducing the AKI rate. These interventions include improving patient conditions, avoiding the use of contrast agents before CPB, avoiding any decrease in the hemoglobin concentration, reasonably using hemodilution, improving oxygen delivery, and controlling the surgery time.

## 3. Renal Replacement Therapy

A meta-analysis of cohort studies reported that RRT is administered to 2.1% of patients with CPB-associated AKI [7]. According to another study, 2.2% of patients required long-term renal support [21]. The early institution of both peritoneal dialysis (PD) for AKI and low cardiac output after cardiac operations removes fluid, thus easing the fluid restriction and improving cardiopulmonary function [72]. In infants at high risk of developing AKI, PD catheter placement has also been shown to be safe, and it is associated with an earlier negative fluid balance, earlier extubation, improved inotrope scores, fewer electrolyte imbalances requiring correction, and improved clinical outcomes [73]. In general, dialysis-associated complications have not been observed during PD [24, 74]. When PD was contraindicated, the use of two small single-lumen catheters in separate veins enables consistent and effective hemodiafiltration in neonates and infants with challenging vascular access, allowing an excellent normalization of the blood flow of metabolic derangements and significant fluid removal [75].

In addition to PD, continuous RRT (CRRT) and hemodialysis (HD) are other suitable RRTs for the treatment of AKI [76]. CRRT is a safe and effective method for fluid and electrolyte homeostasis that allows hyperalimentation in infants and children after cardiac operations [77]. According to a multivariate analysis, intraoperative CRRT preserves postoperative renal function in patients with moderate renal dysfunction before surgery (OR, 0.8; 95% CI, 0.71-0.99; *P* = 0.02) [33]. However, the utilization of CRRT is limited by its high cost. As shown in the study by Sugahara and Suzuki [78], the early initiation of HD therapy (as soon as the urine output decreased to <30 mL/hour) might increase the survival of patients with AKI following cardiac surgery, which was obviously superior to the late-HD group (*P* < 0.01). In addition, perioperative prophylactic HD also decreases operative mortality and morbidity rates in high-risk patients [49]. Furthermore, modern CRRT and HD machines are equipped with exact volumetric systems that direct fluid removal and online solute clearance monitoring, providing obvious superiority and improving physician “comfort” compared with PD that contributes to potentially unpredictable fluid removal rates and possible inadequate solute clearances [76]. Notably, due to the hemodynamic instability of children, RRT has been improved to reduce the cost and enhance the therapeutic effects on neonatal populations (this issue has been discussed in another excellent review [79]).

The early institution of ultrafiltration in the operating room and RRT during the postoperative period may decrease the activity of the proinflammatory milieu and the resulting systemic effects [80]. The early initiation of RRT may prevent fluid overload and result in improved infant outcomes. Despite the theoretical advantages of using RRT and the effective control of uremia, the mortality associated with AKI following CPB remains high, and it is most likely determined by the number of failed organ systems [81]. Thus, the management of CPB-associated AKI should be aimed at relying on more comprehensive interventions.

## 4. Other Interventions

The early mortality of patients utilizing RRT is still very high (RR, 5.3; 95% CI, 3.4-8.1) [7]. Combinations of more effective interventions with RRT are urgently needed to prevent and manage CPB-associated AKI.

Statin therapy is reported to be effective at reducing the risk of contrast-induced AKI [82–84]. Several studies have focused on the effects of statins on CPB-associated AKI. Interestingly, in these studies, patients treated postoperatively with statins showed a significant reduction in sCr levels. However, some studies did not show an association between preoperative statin usage and a decreased incidence of AKI in adults who underwent surgery that required CPB [6, 85]. A recent meta-analysis by Wang and colleagues [86] revealed that preoperative statin therapy reduced the incidence of postoperative AKI by 13% and 7% in subgroups of patients whose AKI was evaluated using the AKIN or the RIFLE criteria, respectively, without significant heterogeneity. Collectively, statins might be a promising therapy for reducing renal complications and the incidence of AKI in patients undergoing CPB surgery [87], and this topic warrants further investigation.

Postoperative AKI has been shown to be associated with the increased intraoperative release of hemeprotein [88, 89]. Hemeprotein release following a mechanical injury is inevitable, but the association between increased plasma free hemoglobin (fHb) levels and renal injury provides new insights into the pathophysiology of AKI [89, 90].

## 5. Further Perspectives

The incidence and prognosis of CPB-associated AKI are influenced by multiple factors. Hence, predictive risk models must be established to comprehensively assess the general condition of patients [91, 92]. Several predictive risk models have been established, including the use of Mehta scores with the C statistic of 0.83 (10 variables: preoperative sCr level, age, race, type of surgery, diabetes, shock, New York Heart Association class, lung disease, recent myocardial infarction, and prior cardiovascular surgery are included in this bedside tool that is aimed at evaluating the need for postoperative dialysis) [93], Cleveland Clinic scores with an overall area under the receiver operating characteristic (ROC) curve of 0.81 (10 variables have been validated for a maximum score of 17: female gender, congestive heart failure, LVEF, preoperative use of intra-aortic balloon pump, COPD, diabetes, previous cardiac surgery, emergency surgery, type of surgery, and preoperative creatinine level) [94], and Simplified Renal Index scores with an overall area under the ROC curve of 0.81 (7 variables are identified for a maximum score of 8: GFR, diabetes, ejection fraction, previous cardiac surgery, procedure other than coronary artery bypass grafting, intra-aortic balloon pump, and nonelective case) [95]. Among these three models, the Cleveland scoring system offers the best discriminative value for predicting postoperative RRT and covers most patients undergoing CPB surgery [96]. It can also be used to predict the composite end point for severe AKI, which enables its broader application in patients at risk of postoperative AKI. Some other predictive scoring systems exist for postoperative RRT [97–99], but these scales have limitations in assessing CPB-associated AKI. Some other models have also been established to estimate CPB-associated AKI but are limited by their use of small derivation cohorts [100, 101]. The addition of novel biomarkers, particularly when used in combinations, significantly increased the predictive value of the model in another study [10]. The ability to accurately assess the risk of developing CPB-associated AKI before surgery should improve clinical management, lead to the earlier involvement of specialist services, and allow more informed decision making; however, further studies are needed to develop a more comprehensive and accurate assessment system.

While the sCr level, which is accepted as a delayed marker of AKI, remains within normal limits [102], other biomarkers are able to identify tubular and glomerular damage. The urine and serum biomarkers for CPB-associated AKI have been separately summarized in Tables 2 and 3. Combinations of two or more of these biomarkers may provide increased diagnostic sensitivity and specificity for evaluating AKI following CPB. Urine neutrophil gelatinase-associated lipocalin (NGAL), interleukin-18 (IL-18), liver fatty acid-binding protein (L-FABP), and kidney injury molecule- (KIM-) 1 levels are sequential predictive biomarkers of AKI that correlate with disease severity and clinical outcomes in pediatric patients who undergo CPB [103, 104]. These biomarkers, particularly when used in combination, may help to establish the timing of injury and allow earlier intervention for AKI [10, 105]. The currently available studies included only limited urinary biomarkers without including other promising biomarkers, such as cystatin C, asymmetric dimethylarginine (ADMA), and NGAL [106]. Thus, a comprehensive “panel” of promising biomarkers that can be used individually and in combination should be developed to optimize both the sensitivity and specificity of predicting and diagnosing CPB-associated AKI. Parikh et al. [107] also proposed that a combination including NGAL and IL-18 might be used for the reliable early diagnosis and prognosis of AKI at all time points after CPB and substantially before an increase in sCr levels is considered to be diagnostic. Some novel predictors, such as elevated levels of plasma renin and IL-8, were recently shown to be associated with development of CPB-associated AKI [108–110], whereas elevated concentration of the serum macrophage migration inhibitory factor is associated with decreased risk of CPB-associated AKI [111]. These factors could be further included in these predictive models. Collectively, a more comprehensive examination of the relevant novel biomarkers both individually and in combination is urgently needed.

**Figure 2:**
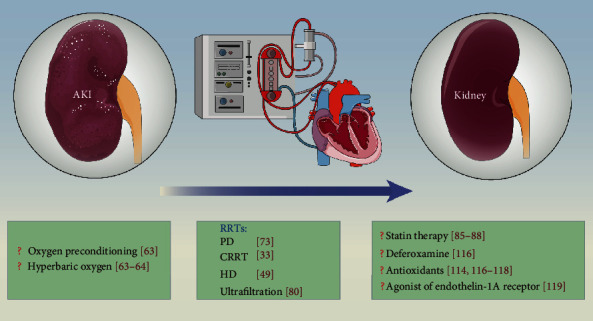
Potential interventions for CPB-associated AKI. RRT is efficient at managing CPB-associated AKI, and other potential interventions may further benefit patients undergoing CPB surgery. AKI: acute kidney injury; RRT: renal replacement therapy; PD: peritoneal dialysis; CRRT: continuous renal replacement therapy; HD: hemodialysis.

Novel biomarkers indicate that free iron-mediated toxicity is an important mechanism of AKI in patients receiving cardiac surgery with CPB [112]. Haase et al. [113] analyzed the pathophysiological implications of some novel renal biomarkers in relation to CPB-associated AKI and found that NGAL, L-FABP, and alpha-1 microglobulin predict the development of CPB-associated AKI, while hepcidin isoforms appeared to predict protection from AKI [114]. However, all of these biomarkers are involved in iron metabolism. A free iron-related, reactive oxygen species-mediated type of kidney injury appears to be the unifying pathophysiological connection between these biomarkers. The effects of deferoxamine, a sequestering agent used to complex iron ions, when used in combination with N-acetylcysteine was inferior to the use of N-acetylcysteine alone in treating gentamicin-induced AKI in adult male Wistar rats [115]. Further studies are needed to determine whether deferoxamine improves the control of AKI following CPB. Billings et al. [88] reported an association between postoperative AKI and both increased intraoperative hemeprotein release and increased lipid peroxidation, indicating a potential role for hemeprotein-induced oxidative damage in the pathogenesis of postoperative AKI. The role of excessive oxidative stress-induced AKI has been widely recognized, and it has been ameliorated by the application of antioxidants [113, 115–117]. Thus, approaches aimed at inhibiting excessive oxidative stress may be an attractive strategy for preventing CPB-associated AKI. CPB-associated AKI is associated with endothelial dysfunction, regional tissue hypoxia, and proximal tubular epithelial cell stress [118]. Antagonism of the endothelin-1A receptor reversed these changes and may therefore represent a therapeutic target for strategies aimed at preventing AKI after CPB surgery (Figure 2).

In a retrospective study, after adjusting for covariates and propensity scores, a multivariate analysis showed that off-pump surgery preserved postoperative renal function in patients with moderate renal dysfunction before surgery (OR, 0.9; 95% CI, 0.87-0.99; *P* = 0.04) [33]. A difference between off-pump and on-pump coronary artery bypass graft surgery was identified in a randomized clinical trial [119]. The off-pump surgery reduced the risk of postoperative AKI by 17%, but the authors did not provide evidence for improved preservation of renal functions at 1 year. Other studies have also supported the hypothesis that off-pump coronary artery bypass graft surgery reduces the risk of postoperative AKI [120, 121]. Based on these studies, interventions are required to reduce the risk of mild to moderate AKI following CPB without altering longer-term renal functions.

## 6. Conclusions

CPB-associated AKI remains a challenging problem with numerous risk factors, including age (>70 years), female gender, smoking history, left ventricular ejection fraction of <35%, borderline renal function, increased sCr levels around the perioperative period, decreased hemoglobin concentrations, hemodilution, and genetic susceptibility (Figure 1 and Table 1). Improvements in our understanding of these factors and establishing models predicting mortality would be beneficial for strategies aimed at predicting and preventing CPB-associated AKI. Some novel biomarkers that may be used to predict and diagnose AKI have attracted attention, and combinations of these biomarkers, including urine (Table 2) and serum biomarkers (Table 3), may provide additional value. The development of a comprehensive “panel” of these risk factors and biomarkers would be useful. The importance and beneficial effect of using RRT for AKI after CPB was validated. Other interventions are needed to assist and increase the effects of RRT (Figure 2). The role of statins in preventing AKI in patients undergoing CPB surgery shows promise and should be further investigated. Furthermore, several studies have investigated the possible mechanisms underlying CPB-associated AKI and found that fHb, free iron, excess oxidative stress, and endothelial dysfunction may be new therapeutic targets for reducing the incidence of AKI and improving the clinical outcomes of patients after CPB surgery.

## Figures and Tables

**Table 1:** Risk factors for acute renal dysfunction following cardiopulmonary bypass.

Factors	Significance	References
Age > 70	An independent risk factor for AKI following CPB	[32, 33]
Female	An independent risk factor for AKI following CPB	[34]
Smoking	An independent risk factor for AKI following CPB	[34]
LVEF < 35%	An independent risk factor for AKI following CPB	[33]
Congenital heart disease	Children with congenital heart disease are at high risk of AKI happening	[36]
eGFR < 60 mL/min/1.73 m^2^	An independent risk factor for AKI following CPB	[38–42]
sCr > 2.5 mg/dL	An independent risk factor for AKI following CPB	[46, 47]
Genetic polymorphisms	Patients with the rs1617640 TT risk allele are more likely to develop AKI following CPB	[51]
Hemoglobin level < 9 g/dL	An independent risk factor for AKI following CPB	[11]
Hemodilution to hematocrit < 24%	An independent risk factor for AKI following CPB	[37]
Oxygen delivery	Increasing oxygen might be protective against AKI following CPB	[57]
During 24 h of exposure to contrast agents	Restricting angiographic examination on the day of operation reduced the AKI rate	[67]
High dose of contrast agent	An independent risk factor for AKI following CPB	[67]
Prolonged surgery duration	Prolonged time on CPB might be associated with increased risk of developing AKI following CPB	[71]

LVEF: left ventricular ejection fraction; eGFR: estimated glomerular filtration rate; sCr: serum creatinine.

**Table 2:** Summary of urine biomarkers for CPB-associated AKI.

Novel biomarkers	Significance	Reference
Urine NGAL	Predictive time point of NGAL for CPB-associated AKI can be advanced as early as 2 h postoperatively.	[10, 107, 122]
Urine IL-18	Urine IL-18 increased at 4-6 h after CPB, peaked at over 25-folds at 12 h, and remained markedly elevated up to 48 h in AKI patients after CPB.	[107]
Urine AIM	Urinary AIM peaks within 2 hours in children who developed AKI after CPB surgery.	[102]
Urine AAG	Urinary AAG peaks within 2 hours in children who developed AKI after CPB surgery.	[102]
Urine Alb	Urinary Alb peaks within 2 hours in children who developed AKI after CPB surgery.	[102]
Urine NAG	During surgery urinary excretion of NAG increased in patients with AKI, reaching peak levels at 15 min after reperfusion.	[89]
Urine hepcidin-25	Elevated urinary hepcidin-25 at 24 h is a strong predictor of avoidance of AKI beyond postoperative day 1.	[123]
Urine TIMP-2	Urine TIMP-2 and IGFBP7 are significantly higher in patients with AKI 1 hour after CPB start.	[124]
Urine IGFBP7		
Urine vasopressinase activity	For patients undergoing CPB surgery, their urine vasopressinase activity peaks at the time of arrival to the ICU. In patients who were diagnosed with AKI, urine vasopressinase activity peaked 30 minutes into CPB.	[125]

NGAL: neutrophil gelatinase-associated lipocalin; IL-18: interleukin-18; AIM: *α*(1)-microglobulin; AAG: *α*(1)-acid glycoprotein; Alb: albumin; NAG: N-acetyl-beta-D-glucosaminidase.

**Table 3:** Summary of serum biomarkers for CPB-associated AKI.

Novel biomarkers	Significance	Reference
Serum cystatin C	The 12 h cystatin C strongly correlated with severity and duration of AKI as well as length of hospital stay. In multivariable analysis, 12 h cystatin C remained a powerful independent predictor of AKI. The discriminatory capacity of plasma cystatin C measured preoperatively and 2 hours after the conclusion of CPB was modest.	[126]
Serum ADMA	Patients with elevated ADMA before surgery were more likely to have prolonged mechanical ventilation, develop LCOS, require an extended length of stay, and require reoperation. ADMA levels inversely correlated with eGFR, but did not predict AKI. Preoperative serum ADMA appears to identify pediatric cardiac surgery patients at risk of poor postoperative outcomes following CPB.	[127]
Plasma fHb	During surgery, plasma fHb increased in patients with AKI, reaching peak levels at 2 h after reperfusion.	[89]
Serum vasopressinase activity	For patients undergoing CPB surgery, their serum vasopressinase activity peaks at the time of arrival to the ICU. In patients who were diagnosed with AKI, serum vasopressinase activity peaked 30 minutes into CPB.	[125]
Plasma micro-RNAs and microvesicle	A number of micro-RNAs (miR-192-5p, miR-487a-3p, miR-490-3p, and miR-501-3p) and microvesicles were differentially expressed in AKI children at 6–12 h following CPB, which might serve as tools for stratification of children at risk of AKI.	[128]
Serum miR-494	Serum level of miR-494 in the death group due to AKI was more than fourfold higher than that in the survival group. Furthermore, its level was identified as an independent risk factor for death due to CPB-associated AKI.	[129]
Serum NGAL	The serum levels of NGAL in the death group were significantly higher than those in the survival group. Furthermore, the expression level of serum NGAL was positively correlated with that of miR-494 in the death group.	[129]
Serum KIM-1	The serum levels of KIM-1 in the death group were significantly higher than those in the survival group. The expression level of serum KIM-1 was positively correlated with that of miR-494 in the death group.	[129]

ADMA: asymmetrical dimethylarginine; LCOS: low cardiac output syndrome; eGFR: estimated glomerular filtration rate; fHb: free hemoglobin; NGAL: neutrophil gelatinase-associated lipocalin; KIM: kidney injury molecule.

## Data Availability

None.
